# Exploring the differential functions of circulating follicular helper T and peripheral helper T cells in rheumatoid arthritis based on metabolism patterns

**DOI:** 10.3389/fimmu.2025.1608675

**Published:** 2025-06-17

**Authors:** Ziran Bai, Siwen Yang, Jinyi Ren, Cheng Zhang, Xianmei Chen, Huina Huang, Guan Wang, Yawei Tang, Jingjing Qi, Xia Li

**Affiliations:** ^1^ Department of Flow Cytometry Center, the Second Affiliated Hospital of Dalian Medical University, Dalian, Liaoning, China; ^2^ Department of Immunology, College of Basic Medical Science, Dalian Medical University, Dalian, Liaoning, China

**Keywords:** rheumatoid arthritis, Tfh cells, Tph cells, glycolysis, mtROS

## Abstract

**Introduction:**

The number of circulating follicular helper T (cTfh) and peripheral helper T (Tph) cells is elevated in rheumatoid arthritis (RA), yet the molecular mechanisms mediating their specific contributions to RA pathology remain unclear. In this study, we explored the distinct function of cTfh and Tph cells based on metabolism patterns in RA.

**Methods:**

Peripheral CD4+ T cells from RA patients were treated with CXCL13 or CCL2, glycolysis inhibitor 2-DG or mitochondria-targeted antioxidant MitoQ *in vitro*. Collagen induced arthritis (CIA) mice were treated with 2-DG or MitoQ *in vivo*. The frequency, transcription factors, functional molecules, cellular senescence, glycolytic activity and mitochondrial ROS (mtROS) of cTfh and Tph cells were assessed. Joint inflammation, CD4+PD-1+ T cells, glycolytic enzymes or IL-1β and IL-6 in ankle joints of CIA mice were detected.

**Results:**

We found that in RA patients, in comparison with Tph cells, cTfh cells show higher levels of Bcl6 and BATF, B helper-related molecules, and glycolytic activity. While Tph cells exhibit higher levels of Blimp1 and T-bet, cytotoxicity-related molecules and mtROS, and more significant cellular senescence characteristics. In addition, CXCL13, the ligand for CXCR5, increases the expression of key glycolytic enzymes in RA cTfh cells, while CCL2 increases mtROS in RA Tph cells. 2-DG reduces the expression of B helper-related molecules cells, and MitoQ mitigates cytotoxic activity of cTfh and Tph cells. Both treatments ameliorate RA symptoms and decrease the number of cTfh and Tph cells in CIA mice.

**Conclusion:**

Our study suggests that in RA patients, cTfh cells display a more robust B helper-associated function, potentially linked to the CXCL13-CXCR5 axis enhancing glycolysis. Tph cells, on the other hand, show greater cytotoxic activity, possibly due to the CCL2-CCR2 axis increasing mtROS production. Targeting glycolysis or mtROS may offer a novel therapeutic strategy for RA patients.

## Introduction

Rheumatoid arthritis (RA) is the most common systematic autoimmune disease affecting approximately 1 of every 200 adults worldwide. It is typically characterized by chronic inflammation in synovial joint, which may cause cartilage and bone destruction, and eventually leads to disability in patients (1, 2). The etiology of RA is not clear, but it is generally believed that certain genetic and environmental risk factors are associated with the development of RA. Diverse immune cells and signals are involved in initiating and sustaining the disorder. Autoantibodies against altered auto-proteins including rheumatoid factor (RF) and anti-cyclic citrullinated peptide (anti-CCP) antibody play an essential role in RA pathogenesis by activating osteoclasts, ultimately leading to joint destruction (3). Follicular helper T (Tfh) cells is a CD4^+^ T cell subset that specializes in providing help to B cell proliferation and differentiation into plasma cells to produce antibodies, and they are critically involved in the pathogenesis of a range of autoimmune diseases, including RA (4).

Tfh cells is a heterogeneous subset characterized by expressing CXCR5, ICOS and PD-1 in germinal center and peripheral blood (5). Our previous studies found that the percentages of circulating Tfh (cTfh) cells were increased and positively correlated with the disease activity and serum anti-CCP antibody levels of RA patients. cTfh cells from RA patients promoted B cells to differentiate into plasma cells to produce antibodies (6, 7). Recent studies have identified an additional PD-1^hi^CXCR5^-^ peripheral helper T (Tph) cells that can help B cells within pathologically inflamed non-lymphoid tissues. Tph cells were increased in inflamed RA joints and peripheral blood of seropositive RA patients (8–10). Sharing similar differentiation mechanisms, both Tfh and Tph cells are key mediators of RA pathogenesis by inducing plasma cell differentiation via IL-21 and SLAMF5 (8, 11–15). However, studies found that only Tfh cells could induce naive B cell differentiation (10), and Tph cells exhibited cytotoxicity by producing cytotoxic molecules such as perforin, granzymes, and G protein-coupled receptor 56 (16, 17). Thus, the exact pathogenetic features of these two circulating PD-1^hi^CD4^+^ T cell populations in RA still need to be explored further.

Glycolysis and mitochondrial metabolism have been shown to be responsible for Tfh cell differentiation and functions (18). Specifically, we have shown that Iguratimod significantly restrained the RA-cTfh cell functions by inhibiting HIF1α-HK2 axis mediated glucose metabolism (6). Choi and colleagues found that glycolysis inhibitor 2-DG treatment suppressed Tfh cell expansion in lupus mice (19). While the role of cellular metabolism in Tph cell functions remains unknown. In this study, we detected the proportions of cTfh and Tph cells and compared the levels of functional molecules and glucose metabolism patterns between these two distinct populations in RA patients. Our findings reveal that both cTfh and Tph cells are increased in RA patients. The cTfh cells demonstrate a stronger capacity to assist B cells, while Tph cells display heightened cytotoxicity, potentially due to enhanced glycolysis in cTfh cells and increased mitochondrial ROS (mtROS) production in Tph cells, respectively. Thus, our study has uncovered specific metabolic programs that regulate the expression of distinct proinflammatory effectors in cTfh and Tph cells of RA patients. These findings could offer new insights into understanding the pathogenesis of RA and inform therapeutic strategies not only for RA but also for other autoimmune diseases.

## Materials and methods

### Patients and healthy controls

Peripheral blood from RA patients and age and gender-matched healthy controls (HC) were obtained from the Department of Rheumatology and Immunology of the Second Affiliated Hospital of Dalian Medical University in China. Detailed clinical characteristics and laboratory features of RA patients and HC are shown in **Table 1**. All RA patients in this study fulfilled the American College of Rheumatology (ACR) 1987 revised criteria. Ethics approval was given by the ethics committee of the Second Hospital of Dalian Medical University (2023-253).

**Table 1:** Clinical and laboratory characteristics of RA patients for study.

Features	RA	HC
Age (years)	58.90 ± 11.54	52.35±11.08
Male/Female	6/34	4/24
RF (/mL)	187.3 ± 275.0	–
CRP (mg/L)	16.61 ± 17.73	–
Anti-CCP (U/mL)	133.20 ± 73.42	–
ESR (mm/h)	44.08 ± 20.23	–

RF, rheumatoid factor; CRP, C-reactive protein; anti-CCP, anti-cyclic citrullinated peptide antibodies; ESR, erythrocyte sedimentation rate.

### Collagen-induced arthritis model

Male DBA/1J mice aged 6–8 weeks were purchased from Shanghai SLAC Laboratory Animals Company and housed under pathogen-free conditions at the Laboratory Animal Center of Dalian Medical University. For the primary immunization, mice were subcutaneously injected in the tail with the emulsion formed by type II bovine collagen (Chondrex, USA) and Complete Freund adjuvant (CFA, Chondrex). Administer a booster injection consisting of type II bovine collagen and Incomplete Freund adjuvant (IFA, Chondrex) emulsion on day 21. The injection consisted of 100 µL of an emulsion containing 100 µg of collagen and 2 mg/mL of CFA or IFA.

The study covered the healthy control mice group (n=5), the CIA mice group (CIA mice were treated with vehicle, n=5), and the CIA+2-DG mice group (CIA mice were treated with 2-DG three times a week for 2 weeks on day 28 after immunization, 500 mg/kg, n=5), and the CIA+MitoQ mice group (CIA mice were treated with MitoQ three times a week for 2 weeks on day 28 after immunization, 2 mg/kg, n=5). The mice’s paw thickness and disease activity score were assessed. Disease activity scores were derived from the evaluation of clinical arthritis in all four limbs as reported by the scoring system for the evaluation of arthritis severity. The scoring system was defined as 0, no evidence of erythema and swelling; 1, erythema and mild swelling confined to the tarsals or ankle joint; 2, erythema and mild swelling extending from the ankle to the tarsals; 3, erythema and moderate swelling extending from the ankle to the metatarsal joints; and 4, erythema and severe swelling encompassing the ankle, foot and digits or ankylosis of the limb. All experiments complied with the guidelines established by the Institutional Animal Care and Use Committee (IACUC). The study was approved by the Ethics Committee of the Dalian Medical University (Approval No: AEE24013).

### Histology

The mice’s ankle joints and paws were fixed overnight in 4% paraformaldehyde, decalcified in 14% EDTA, and embedded in paraffin. Sections of 5-μm thickness were generated from the paraffin tissue blocks. And the sections were stained with hematoxylin-eosin (HE, Solarbio, China), and Safranin O-fast green (SO/FG, Solarbio, China). The expression of hexokinase 2 (HK2, Cell Signaling Technology), lactate dehydrogenase (LDH, Cell Signaling Technology), IL-1β (Affinity), and IL-6 (Affinity) in synovium were analyzed by using immunohistochemistry. The sections were incubated with CD4-FITC (Biolegend) and PD-1-PE (Biolegend) antibodies in a 37°C wet box for 1 h, and the images were scanned under fluorescence microscopy.

### Flow cytometry

For cell surface staining, cells were stained in PBS with Biolegend or eBioscience antibodies (Fixable viability dye (FVD), anti-CD4, anti-CXCR5, anti-PD-1, anti-ICOS, anti-CD40L, anti-CD27, anti-CD28, anti-CCR7, anti-CD107a and anti-Glut1 antibodies) for 20 min.

For intracellular staining of Perforin and Granzyme B, peripheral blood mononuclear cells (PBMCs) were fixed and permeabilized by Cytofix/Cytoperm Intracellular Staining Kit (BD Biosciences) for 60 min after cell surface staining, and labeled with anti-Perforin and anti-Granzyme B antibodies for 30 min.

For intracellular cytokines staining, cells were incubated with 50 ng/mL phorbol myristate acetate (PMA), 1 μg/mL Ionomycin, and 10 μg/mL Brefeldin-A (BFA) for 4 hours before staining with surface antibodies. Then cells were fixed and permeabilized to stain with cytokines antibodies (anti-IL-21, anti-CXCL13, anti-IL-4, anti-IFN-γ, and anti-TNF-α antibodies) for 30 min.

For transcription factor staining, cells were fixed and permeabilized using the Transcription Factor Fixation/Permeabilization Buffer Set (eBioscience) for 45 min. Then cells were stained with anti-BATF, anti-Bcl-6, anti-T-bet, and anti-Eomes.

All stained cells were analyzed on the Flow Cytometer (NovoCyte 2060R) and data were analyzed with NovoExpress software.

### Senescence-associated β-galactosidase staining

Levels of SA-β-Gal activity were assessed by Senescence Assay Kit (Beta Galactosidase, Fluorescence, Abcam). Collect and resuspend cells in 500 µL of fresh media containing 1.5 µL of Senescence Dye per tube. Cells were incubated for 1 h at 37°C, 5% CO2, then washed twice with 500 µL wash buffer, and stained with cell surface antibodies, and analyzed immediately using flow cytometry.

### Measurement of mitochondrial ROS

Levels of mtROS were assessed by MitoSOX Red probe (Invitrogen). After staining with surface antibodies, cells were stained with 5 μM MitoSOX Red probe in Hanks’ balanced salt solution buffer for 30 min at 37°C. Then wash the cells gently three times for flow cytometry.

### JC-10 staining

Mitochondrial membrane potential (MMP) was determined by Mitochondrial Membrane Potential Kit (JC-10 assay; Solarbio) according to the instruction of the manufacturer. Briefly, after staining with surface antibodies, cells were stained with JC-10 staining solution at 37°C for 20 min. Then wash the cells gently three times with warm Hank’s for flow cytometry.

### Glucose metabolism-related analysis

For glucose uptake assay, PBMCs from RA patients were resuspended and cultured in glucose free RPMI 1640 medium (Gibco) for 30 min and then cultured with 50 μM D-glucose analog 2-[N-(7-nitrobenz-2-oxa-1,3-diazol-4-yl) amino]-2-deoxy-D- glucose (2-NBDG) (Sigma-Aldrich) for 30 min at 37°C. For HK2 staining, the cells were fixed in 4% paraformaldehyde and permeabilized in 0.5% Triton X-100 before anti-HK2 antibody staining. For LDH staining, the cells were fixed in 4% paraformaldehyde and permeabilized in 90% methanol before LDH antibody staining. All stained cells were analyzed by using flow cytometry.

### Cell culture

CD4^+^ T cells (purity range 95%-99%) were purified from PBMCs of RA patients using the Human CD4^+^ T Cell Isolation Kit (BioLegend), and cultured with plate-coated anti-CD3 antibody (5 μg/mL, eBioscience) and anti-CD28 antibody (2 μg/mL, eBioscience) in RMPI-1640 medium containing 10% fetal bovine serum and 1% penicillin/streptomycin for 72 h. In several experiments, CXCL13 (100 μg/mL, Peprotech), CCL2 (100 μg/mL, Peprotech), 2-DG (1 mM, Solarbio) or MitoQ (200 nM, Biovision) was administrated to the cell culture.

### Statistical analysis

Data is presented as means ± standard errors of the mean (SD). GraphPad Prism 9 was used to conduct all statistical analyses. Statistical differences were analyzed by paired t-test, Wilcoxon rank sum test, unpaired t-test, Welch’s t-test, and Mann-Whitney test. Paired t-tests, unpaired t-tests, or Welch t-tests were used to compare parameter data. The Mann-Whitney test or Wilcoxon matched-pairs signed rank test was performed for non-parametric data. The P-values < 0.05 were considered significant. Asterisks mark the significant differences between different groups (*P<0.05; **P<0.01 and ***P<0.001).

## Results

### cTfh and Tph cells accumulate in RA patients and CIA mice

**Figure 1:**
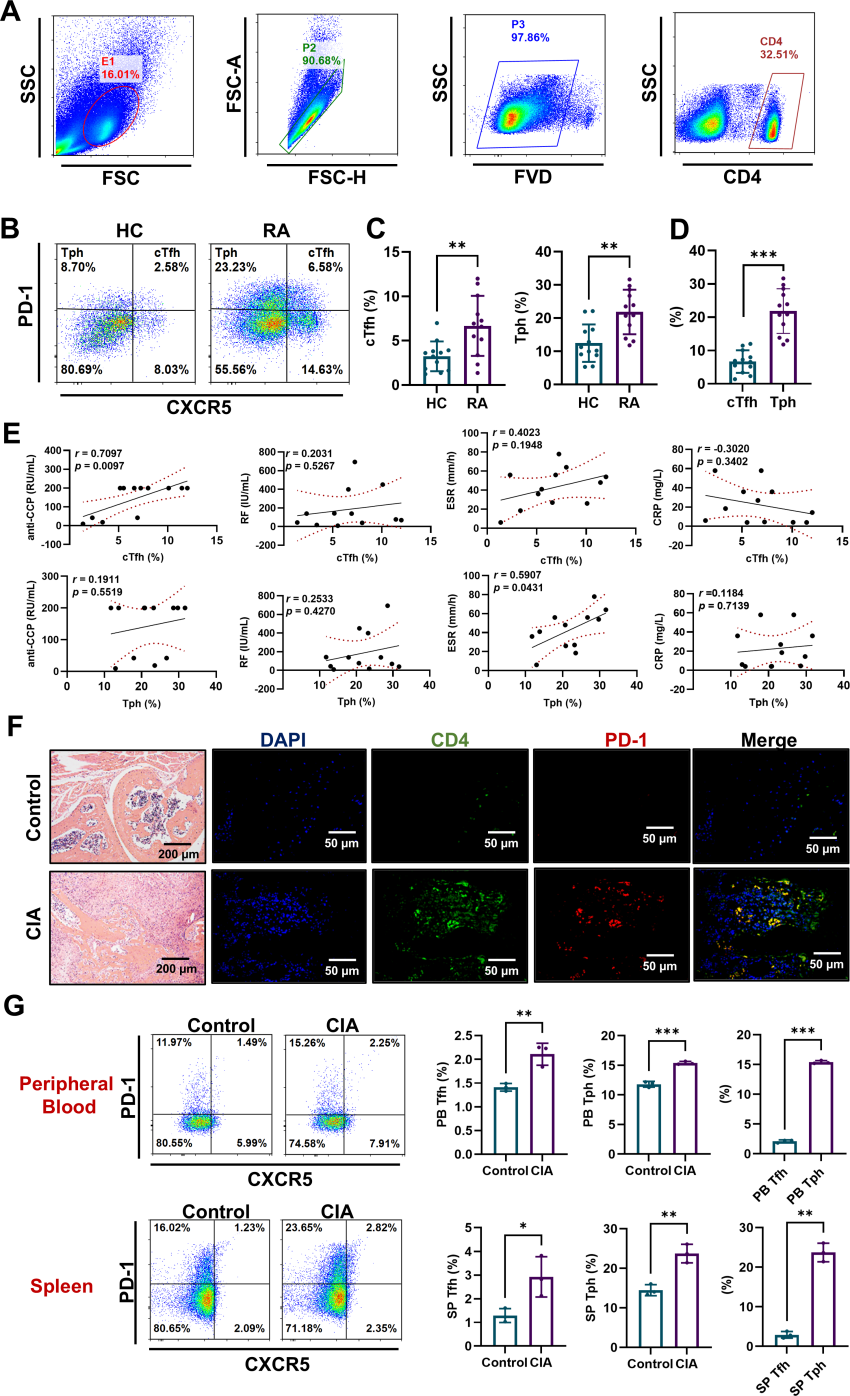
cTfh and Tph cells accumulated in RA patients and CIA mice. **(A-E)** PBMCs were isolated from HC (n=12) and RA patients (n=12). **(A, B)** Flow cytometric gating strategy of CD4^+^CXCR5^+^PD-1^+^ cTfh cells and CD4^+^CXCR5^-^PD-1^+^ Tph cells. **(C)** Comparison of cTfh and Tph cell percentages in HC and RA patients. **(D)** Comparison of cTfh and Tph cell percentages in RA patients. **(E)** Relationships of percentages of cTfh and Tph cells with anti-CCP antibody, RF, ESR and CRP in RA patients. **(F)** CD4^+^PD-1^+^ T cells in ankle joint of CIA mice were detected by immunofluorescence. **(G)** Percentages of Tfh and Tph cells in spleen and peripheral blood of CIA mice n=3 were measured by FCM. *P<0.05; **P<0.01; ***P<0.001.

To delve into the precise pathogenic characteristics of cTfh and Tph cells, we initially determined the percentages of these two T cell subsets in the peripheral blood of HC and RA patients using flow cytometry. CXCR5^+^PD-1^+^ cTfh and CXCR5^-^PD-1^+^ Tph cells were gated from CD4^+^ T cells (**Figures 1A, B**). Results show that RA patients have a higher number of cTfh and Tph cells than HC (**Figure 1C**). And the frequency of Tph cells is higher than that of cTfh cells in RA patients (**Figure 1D**). Further, the percentages of cTfh cells are positively correlated with the levels of serum anti-CCP antibody, and the percentages of Tph cells are positively correlated with erythrocyte sedimentation rate (ESR) in RA patients. No significant correlation is found between the percentages of cTfh or Tph cells and the levels of RF and C-reactive protein (CRP) in RA patients (**Figure 1E**). What’s more, immunofluorescence results show that CD4^+^PD-1^+^ T cells are increased in ankle joint of CIA mice (**Figure 1F**). Compared to control mice, CIA mice show higher levels of Tfh and Tph cells in peripheral blood and spleen, and the frequency of Tph cells is higher than that of cTfh cells (**Figure 1G**). These results indicate that the accumulated cTfh and Tph cells may play distinct roles in the pathogenesis of RA.

### cTfh cells express more B helper-associated functional molecules, while Tph cells express more cytotoxicity-associated molecules

**Figure 2:**
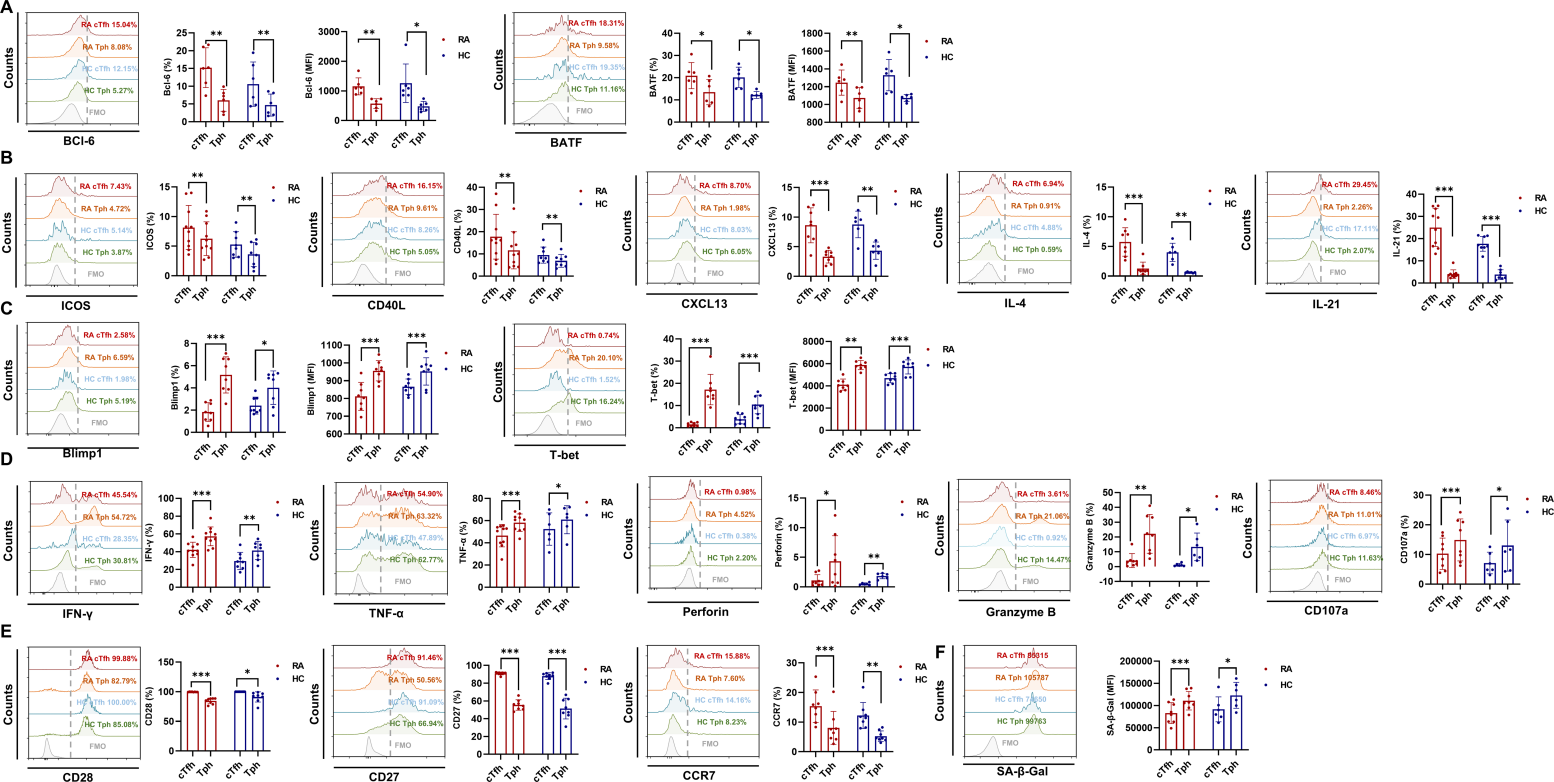
cTfh and Tph cells tend to express different functional molecules in RA patients and HC. **(A-F)** Expression of Bcl6 (RA: n=6; HC: n=6), BATF (RA: n=6; HC: n=6), ICOS (RA: n=10; HC: n=8), CD40L (RA: n=10; HC: n=8), CXCL13 (RA: n=8; HC: n=6), IL-4 (RA: n=8; HC: n=6), IL-21 (RA: n=10; HC: n=8), Blimp1 (RA: n=8; HC: n=8), T-bet (RA: n=8; HC: n=8), IFN-γ (RA: n=10; HC: n=8), TNF-α (RA: n=10; HC: n=6), perforin (RA: n=8; HC: n=6), granzyme B (RA: n=8; HC: n=6), CD107a (RA: n=8; HC: n=6), CD28 (RA: n=8; HC: n=8), CD27 (RA: n=8; HC: n=8) and CCR7 (RA: n=8; HC: n=8), and SA-β-gal activity (RA: n=8; HC: n=6) in cTfh and Tph cells from RA patients and HC were detected by FCM. *P<0.05; **P<0.01; ***P<0.001.

Studies have demonstrated that the transcription factor BATF facilitates Tfh cell differentiation by promoting Bcl6 expression in CD4^+^ T cells (20). ICOS, CD40L, CXCL13, IL-4 and IL-21 of Tfh cells are important for B cell activation and differentiation (8, 21–24). Our results show that compared to Tph cells, cTfh cells express higher levels of Bcl6 and BATF (**Figure 2A**), ICOS, CD40L, CXCL13, IL-4 and IL-21 (**Figure 2B**) in RA patients and HC, which indicate that cTfh cells have a more potent ability for B-cell recruitment and assistance than Tph cells.

In addition to B cell-helping function, Tph cells were reported to exhibit cytotoxic activity (16, 17). Transcription factors Blimp1 and T-bet are important for cytotoxic effector T cell differentiation and function (25). Our results show that compared to cTfh cells, Tph cells express higher levels of Blimp1 and T-bet (**Figure 2C**), IFN-γ, TNF-α, perforin, granzyme B and CD107a (**Figure 2D**) in RA patients and HC. According to reports, senescent T cells are characterized by down-regulation of CCR7, CD28 and CD27, and up-regulation of NK receptors and innate-like killing effects (26, 27). Thus, we detected the cellular senescence biomarkers and cytotoxicity-associated molecules in cTfh and Tph cells of RA patients. Results show that compared to cTfh, Tph cells show lower levels of CD28, CD27 and CCR7 (**Figure 2E**), and higher levels of senescence associated SA-β-gal activity (**Figure 2F**) in RA patients and HC. These results indicate that Tph cells have more pronounced cellular senescence characteristics and stronger cytotoxic activity than cTfh cells.

### Enhanced glycolysis in cTfh cells and elevated mtROS in Tph cells

**Figure 3:**
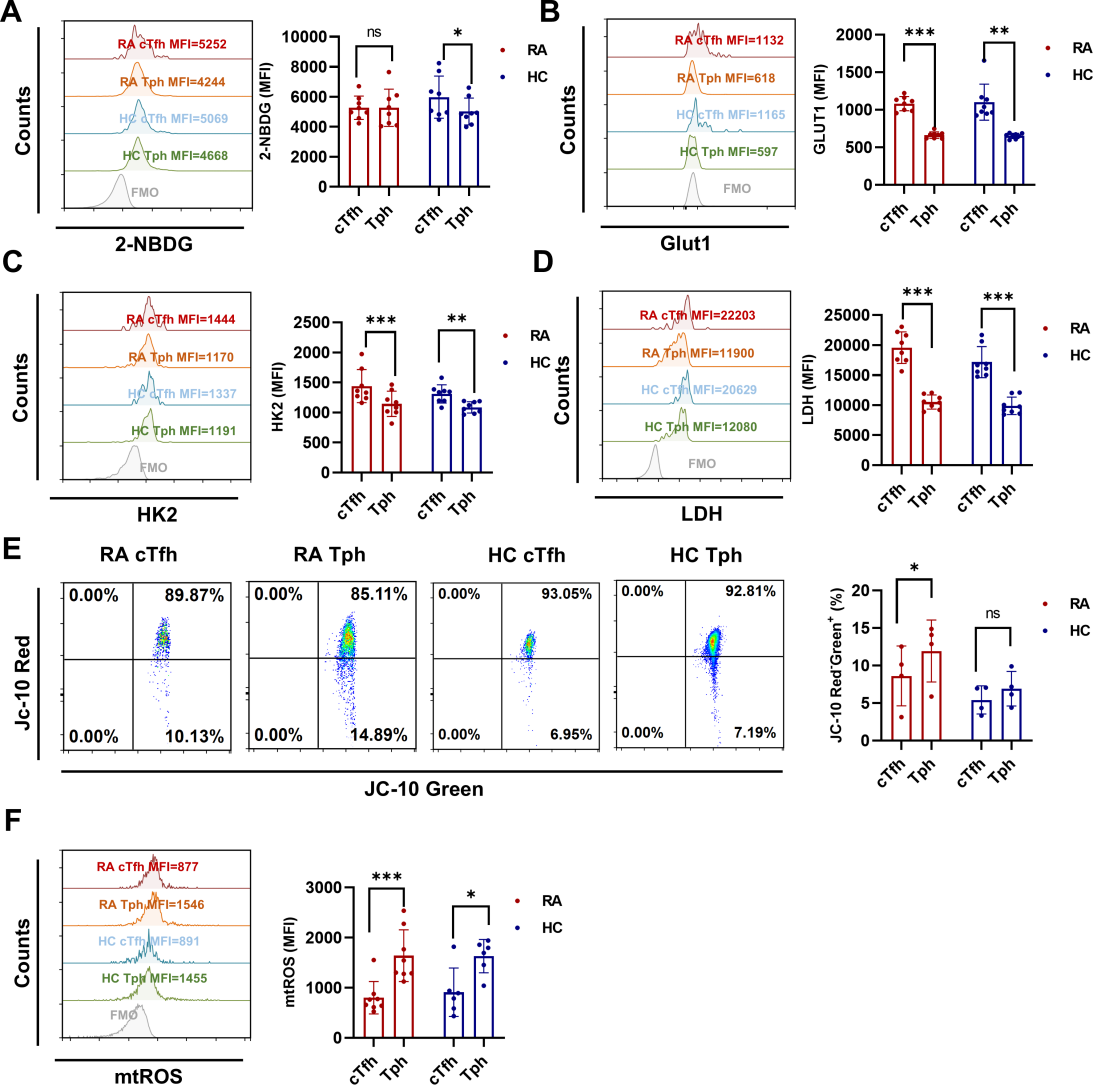
Enhanced glycolysis in cTfh cells and elevated levels of mtROS in Tph cells from RA patients and HC. **(A)** Glucose uptake of cTfh and Tph cells was determined by the 2-NBDG method (RA: n=8; HC: n=8). **(B-D)** Expression levels of GLUT1 (RA: n=8; HC: n=8), HK2 (RA: n=8; HC: n=8), and LDH (RA: n=8; HC: n=8) in cTfh and Tph cells were measured by FCM. **(E)** Mitochondrial membrane potential was determined by JC-10 probes (RA: n=4; HC: n=4). **(F)** mtROS were determined by MitoSOX probes (RA: n=8; HC: n=6). ns, no significance; *P<0.05; **P<0.01; ***P<0.001.

Our previous study found that Iguratimod restrained RA-cTfh cell functions by inhibiting HIF1α-HK2 axis mediated glucose metabolism (6). To explore whether the distinct functions of cTfh and Tph cells are related to cellular glucose metabolism, we detected the glucose uptake ability and the expression of key glycolytic molecules in cTfh and Tph cells of RA patients and HC. Despite these two T cell subsets showing similar glucose uptake ability (**Figure 3A**) in RA patients, cTfh cells express higher levels of glucose transporter 1 (GLUT1), HK2 and LDH than Tph cells (**Figures 3B-D**) in RA patients and HC. These results suggest that cTfh cells exhibit a higher level of glycolysis than Tph cells. Increased ROS production contributes to T cell senescence, and oxidation of NADPH produced by oxidative phosphorylation is one of the main sources of ROS production (28, 29). Given the lower levels of glycolytic molecules observed in Tph cells than cTfh cells, we hypothesized that Tph cell functions are associated with mitochondrial metabolism. In this study, JC-10 and MitoSOX were used to detect mitochondrial function. Results show that Tph cells exhibit higher levels of mitochondrial membrane potential and mtROS (**Figures 3E-F**) in RA patients, which suggest that Tph cells show mitochondrial dysfunction, resulting in elevated levels of mtROS.

### CXCL13-CXCR5 signaling upregulates glycolysis in cTfh cells, while CCL2-CCR2 signaling increases mtROS production in Tph cells of RA patients

**Figure 4:**
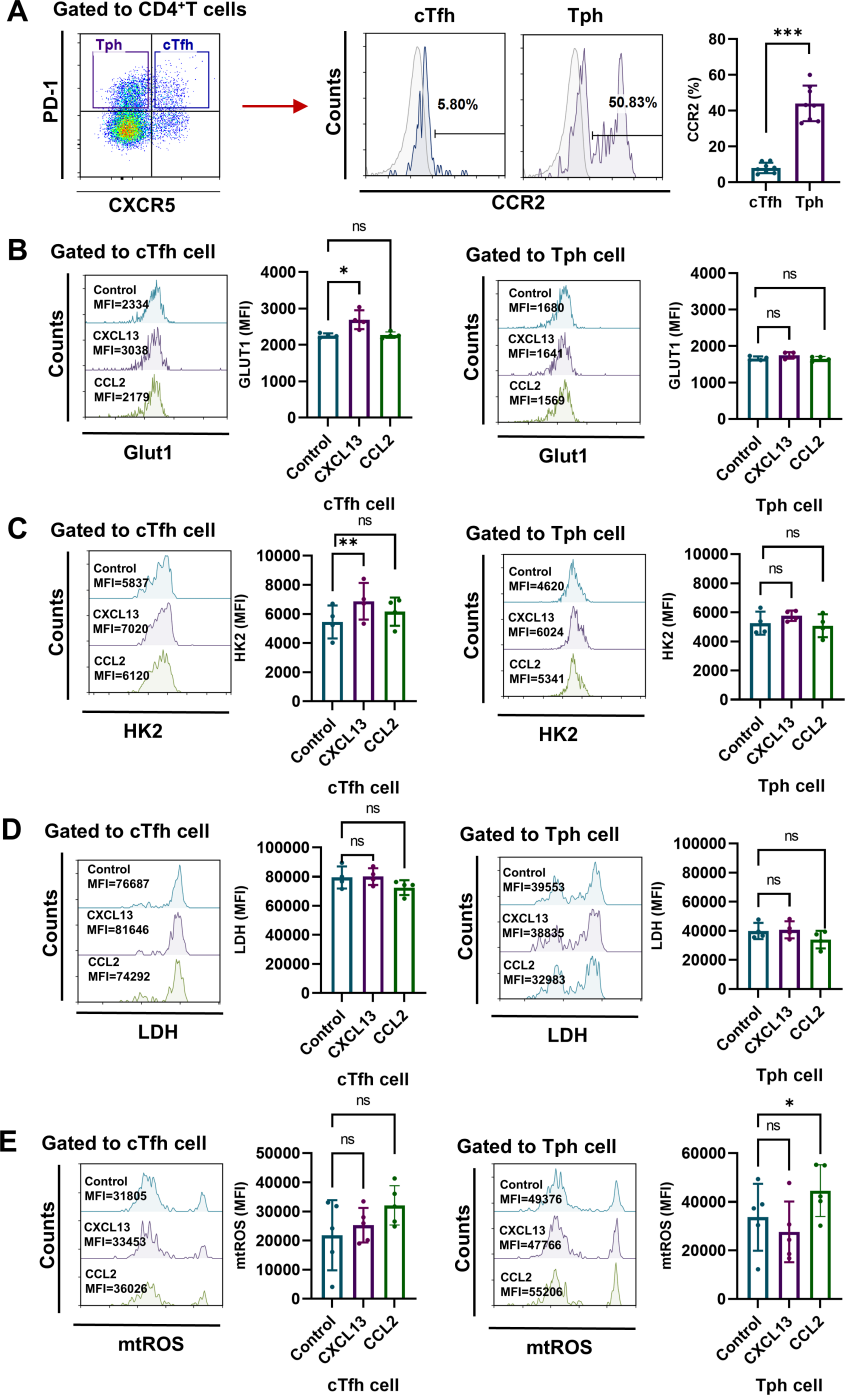
CXCL13-CXCR5 signaling pathway enhances glycolysis in RA cTfh cells, while CCL2-CCR2 signaling pathway increases mtROS production in Tph cells from RA patients. **(A)** Expression levels of CCR2 on CD4^+^CXCR5^+^PD-1^+^ cTfh cells and CD4^+^CXCR5^-^PD-1^+^ Tph cells from RA patients were measured by FCM (n=8). **(B-E)** Peripheral CD4^+^ T cells were isolated from PBMCs of RA patients and stimulated with CXCL13 or CCL2 for 72h. **(B-D)** Expression levels of GLUT1, HK2 and LDH were measured by FCM (n=4). **(E)** Levels of mtROS were determined by MitoSOX probes (n=5). ns, no significance; *P<0.05; **, *P*<0.01; ***P<0.001.

According to reports, despite a lack of CXCR5 expression, Tph cells are characterized by expressing CCR2 (8). Here, our results confirm that CXCR5^-^PD-1^+^ Tph cells expressed higher level of CCR2 than CXCR5^+^PD-1^+^ cTfh cells from RA patients (**Figure 4A**). Next, to verify the distinct metabolism patterns in cTfh and Tph cells of RA patients, we measured the levels of GLUT1, HK2, LDH and mtROS in RA CD4^+^ T cells stimulated with CXCL13 (ligand for CXCR5) or CCL2 (ligand for CCR2). Results show that CXCL13-CXCR5 signaling up-regulate the expression of GLUT1 and HK2 in cTfh cells, while CCL2-CCR2 signaling promote the production of mtROS in Tph cells (**Figures 4B-E**). These results suggest that the enhanced glycolysis in RA-cTfh cells is related to CXCL13-CXCR5 signaling, while the increased mtROS in RA-Tph cells is related to CCL2-CCR2 signaling.

### Inhibition of glycolysis or scavenging of mtROS down-regulated the expression of functional molecules in cTfh and Tph cells of RA patients

**Figure 5:**
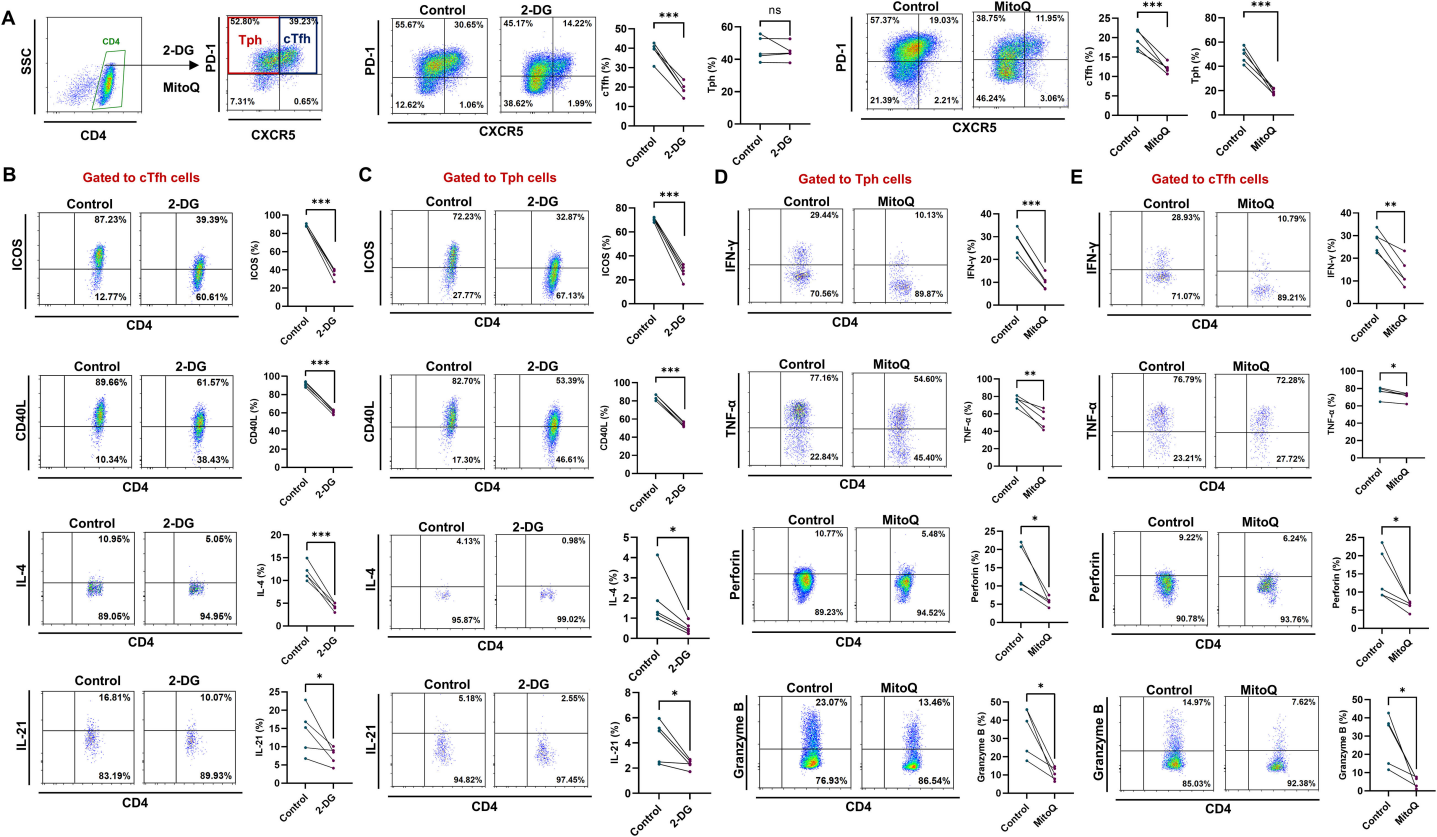
Inhibition of glycolysis or mtROS production leads to the downregulation of functional molecules in cTfh and Tph cells from RA patients. **(A-E)** Purified RA-CD4^+^ T cells were cultured in the presence of 2-DG or MitoQ for 72 h. Expression of ICOS, CD40L, IL-4, IL-21, IFN-γ, TNF-α, perforin and granzyme B in cTfh and Tph cells was detected by FCM (n=5). ns, no significance; *P<0.05; **P<0.01; ***P<0.001.

To further investigate the roles of glycolysis and mtROS in the differential functions of cTfh and Tph cells, we inhibited glycolysis using the glucose analog 2-DG and mitigated mtROS with the mitochondria-targeted antioxidant MitoQ in CD4^+^ T cells from RA patients, we found that 2-DG could reduce the proportion of cTfh, and MitoQ could decrease the proportion of both cTfh and Tph cells (**Figure 5A**). Results show that glycolysis inhibition down-regulate the expression of ICOS, CD40L, IL-4 and IL-21 in both cTfh and Tph cells (**Figures 5B, C**). mtROS scavenging significantly down-regulate the expression of IFN-γ, TNF-α, perforin and granzyme B in both Tph and cTfh cells (**Figures 5D, E**). These results suggest that in RA patients, the enhanced B-cell recruitment and helper functions of cTfh cells may rely on increased glycolysis, while the heightened cytotoxic activity of Tph cells may be associated with elevated mtROS levels.

### Inhibition of glycolysis or scavenging of mtROS alleviated disease severity in CIA mice

**Figure 6:**
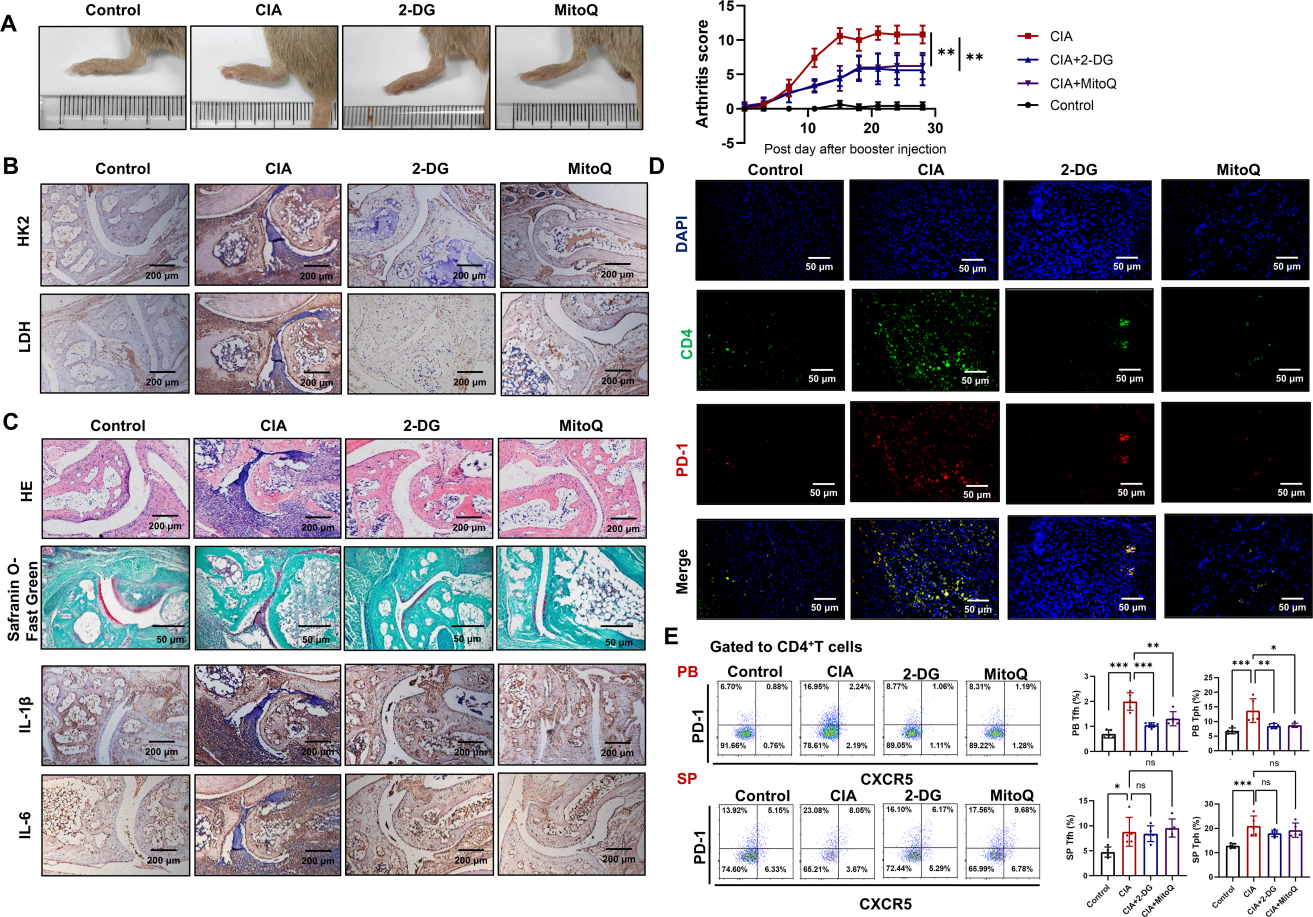
Inhibition of glycolysis or scavenging of mtROS reduces disease severity in CIA mice. CIA mice were treated with 2-DG or MitoQ (n=5). **(A)** Hind paw images and clinical scores of CIA mice. **(B)** The expression of HK2 and LDH in the ankle joints of CIA mice were measured by immunohistochemistry. **(C)** HE and safranin O-fast green staining images of ankle joints (upper two panels). Immunohistochemistry staining images of IL-1β and IL-6 in ankle joints (lower two panels). **(D)** CD4^+^PD-1^+^ T cells in ankle joint were detected by immunofluorescence. **(E)** The percentages of Tfh and Tph cells in peripheral blood and spleen of CIA mice were measured by FCM. ns, no significance; *P<0.05; **P<0.01; ***P<0.001.

To confirm the roles of glycolysis and mtROS in the differential functions of cTfh and Tph cells *in vivo*, we administered 2-DG or MitoQ to CIA mice. Results show that both 2-DG and MitoQ reduce paw swelling and arthritis score (**Figure 6A**), and the expression of HK2 and LDH in ankle joints (**Figure 6B**). Additionally, 2-DG and MitoQ improve the pervasive infiltration of inflammatory cells, cartilage and bone tissue erosion, and synovial hyperplasia (**Figure 6C**, upper two panels). 2-DG and MitoQ also reduce the expression of inflammatory cytokines IL-1β and IL-6 (**Figure 6C**, lower two panels) and the presence of CD4^+^PD-1^+^ T cells in ankle joints (**Figure 6D**). Furthermore, 2-DG and MitoQ down-regulate the percentages of cTfh and Tph cells in the peripheral blood of CIA mice, although not in the spleen (**Figure 6E**). These results suggest that inhibition of glycolysis or mtROS production alleviate disease severity in CIA mice via restraining the development and recruitment of cTfh and Tph cells.

## Discussion

Both cTfh and Tph cells play crucial roles in RA pathogenesis. Our current study discovered that in RA patients, the frequency of CXCR5^+^PD-1^+^ cTfh and CXCR5^-^PD-1^+^ Tph cells is elevated. Further, cTfh cells express higher levels of B helper functional molecules due to enhanced glycolysis, and Tph cells display more pronounced cellular senescence characteristics and higher levels of cytotoxic activity-related molecules due to increased mtROS production in RA patients.

Tfh cells, as a heterogeneous subset of CD4^+^ T cells, may express different phenotypic biomarkers according to cell differentiation stages and disease conditions (4). Researchers have compared the shared and distinct characteristics of Tfh and Tph cells in human diseases (10, 30, 31). Although, Tph cells are mainly present within inflamed non-lymphoid tissues due to expressing CCR2, CCR5 and CX3CR1, and can be distinguished from Tfh cells by the high expression of PD-1 and no expression of CXCR5 (8, 32). In some studies, Tph cells that showed Tfh-associated genes and functions might be mixed with Tfh cells (33, 34). In this study, we found cTfh and Tph cells prefer to express differential functional molecules based on different metabolism patterns, which contribute to elucidating RA pathogenesis further.

Tfh and Tph cells share the expression of the checkpoint molecule PD-1, which is typically expressed on exhausted T cells under continuous antigenic stimulation (35, 36). While, unlike exhausted cytotoxic T lymphocytes, Tfh and Tph cells can cause pathological autoantibody production and tissue injury despite negative signals provided by PD-1 in autoimmune diseases (37, 38). Reports indicate that Tph cells are not increased in seronegative early RA or spondyloarthritis patients, but they are increased and contribute to pathological B cell activation and inflammation in seropositive RA and systemic lupus erythematosus patients (9, 10, 39). After stimulation, Tph cells exhibited Th1-like auto-reactivity by producing IFN-γ, IL-21, TNF-α and CXCL13 in the joints of RA patients, which was regulated by PD-1 signaling (37). However, due to sampling limits and technical difficulties, the function of Tph cells in disease pathogenesis still is not elucidated clearly (8, 40). We observed that cTfh cells tend to express B helper functional molecules, and Tph cells tend to express cytotoxicity-related molecules, indicating distinct mechanisms underlying their pathological roles. Notably, while cTfh and Tph cells exhibited similar functional disparities between RA patients and HC, some functional molecules (such as ICOS, CD40L, CXCL13, IL-21, IFN-γ, perforin, and granzyme B) in RA cTfh and Tph exhibited elevated levels. And the expanded populations of cTfh and Tph cells in RA highlight the imperative to delineate their functional contributions in RA.

Others’ studies revealed the cytotoxic potential of Tph cells in submandibular glands (SMG) of IgG4-related disease (IgG4-RD) and synovial fluid of RA patients (16, 17). The functional assays suggested their specific lysis against vascular endothelial cells and ductal epithelial cells, which might contribute to the lesions in SMG of IgG4-RD (17). Elahee M and colleagues found that in systemic sclerosis patients, among PD-1^high^CXCR5^-^ Tph cells, only the HLA-DR^+^ICOS^-^ cells with cytotoxic properties are expanded and significantly correlated with the severity of interstitial lung disease (41). In our study, we confirmed that Tph cells from RA patients, which express low levels of ICOS, are characterized by the production of cytotoxic molecules such as perforin and granzyme B, a feature not observed in cTfh cells. In addition, Tph cells are divided into four subsets CXCR3^+^CCR6^-^Tph1, CXCR3^-^CCR6^-^Tph2, CXCR3^-^CCR6^+^Tph17, and CXCR3^+^CCR6^+^Tph1–17 cells. Study found that Tph1 and Tph17 cells showed B-helper functions, while Tph2 cells exhibited cytotoxic activity in systemic lupus erythematosus patients (42). Further studies are needed to elucidate the metabolism patterns in different Tph subsets, and functional assays are required to identify the specific target cells of Tph cells in RA patients.

According to reports, PD-1 pathway promotes cancer cell growth by activating downstream mTOR signaling, which is a master regulator of glucose metabolism by promoting HIF-1α expression (43, 44). Transcription factor HIF-1α, in turn, activates the transcription of glucose metabolism-related genes, including GLUT1 and HK, by binding to the hypoxia-response element in their promoters (45). CXCL13 has been shown to activate mTOR signaling pathway through its interaction with CXCR5 on renal cell carcinoma (46). In our study, cTfh cells from RA patients exhibit higher levels of glycolytic molecules GLUT1 and HK2, which may be linked to the CXCL13-CXCR5 axis-mediated mTOR activation. Subsequently, the enhanced glycolysis upregulates the levels of CD40L and ICOS on cTfh cells (47, 48).

The lower levels of glycolytic molecules observed in Tph cells than cTfh cells prompted us to hypothesize that Tph cell functions are associated with mitochondrial metabolism. The level of mtROS is one of the crucial parameters for mitochondrial function and mediators for cellular senescence (49, 50). And CCL2 has been reported to be a crucial mediator for ROS generation in monocyte (51). According to reports, senescent T cells are characterized by up-regulation of NK receptors and innate-like killing effects through the expression of perforin, granzymes and TNF-α (26). Our results show that Tph cells from RA patients exhibit higher levels of mtROS and senescence associated SA-β-gal activity than cTfh cells (**Figures 3F** and **2F**). mtROS scavenging significantly down-regulate the expression of cytotoxicity-associated molecules in Tph cells (**Figures 5D, E**). These results suggest that the high cytotoxic activity might be due to increased mtROS induced cellular senescence in Tph cells. While mitochondria are a major source of ROS generation, the relationship between ROS and cytotoxicity is complex and may involve other factors. Further research is necessary to elucidate the precise mechanisms underlying the interplay between mtROS, cytotoxicity and cellular senescence in Tph cells.

## Conclusion

**Figure 7:**
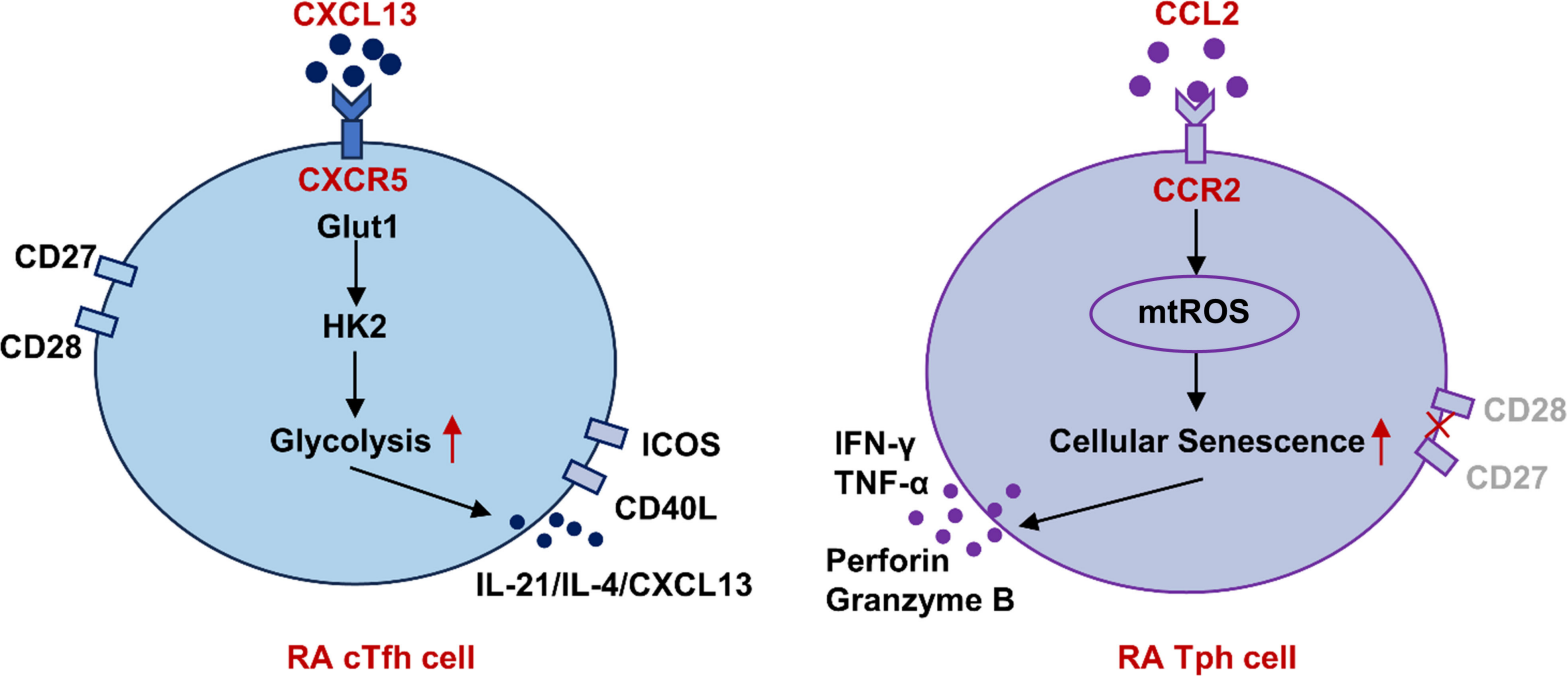
Schematic representation of differential functions of cTfh and Tph cells in RA based on metabolism patterns. In RA patients, cTfh cells display a more potent B helper-associated function, likely due to enhanced glycolysis driven by the CXCL13-CXCR5 axis. Meanwhile, Tph cells exhibit increased cytotoxic activity, which may be linked to elevated mtROS driven by the CCL2-CCR2 axis. RA, rheumatoid arthritis; cTfh, circulating follicular helper T; Tph, peripheral helper T; Glut1, glucose transporter 1; HK, hexokinase; mtROS, mitochondrial reactive oxygen species.

To summarize, in RA patients, cTfh cells display a more potent B helper-associated function, likely due to enhanced glycolysis driven by the CXCL13-CXCR5 axis. Meanwhile, Tph cells exhibit increased cytotoxic activity, which may be linked to elevated mtROS driven by the CCL2-CCR2 axis (**Figure 7**). Targeting glycolysis or mtROS may offer a novel therapeutic strategy for RA patients.

## Data Availability

The raw data supporting the conclusions of this article will be made available by the authors, without undue reservation.

## References

[1] Smith MH Berman JR. What is rheumatoid arthritis? JAMA. (2022) 327:1194. doi: 10.1001/jama.2022.0786 35315883

[2] Gao Y Zhang Y Liu X. Rheumatoid arthritis: pathogenesis and therapeutic advances. MedComm. (2024) 5:e509. doi: 10.1002/mco2.509 38469546 PMC10925489

[3] Babaahmadi M Tayebi B Gholipour NM Kamardi MT Heidari S Baharvand H. Rheumatoid arthritis: the old issue, the new therapeutic approach. Stem Cell Res Ther. (2023) 14:268. doi: 10.1186/s13287-023-03473-7 37741991 PMC10518102

[4] Qi J Liu C Bai Z Li X Yao G. T follicular helper cells and T follicular regulatory cells in autoimmune diseases. Front Immunol. (2023) 14:1178792. doi: 10.3389/fimmu.2023.1178792 37187757 PMC10175690

[5] Song W Craft J. T follicular helper cell heterogeneity. Annu Rev Immunol. (2024) 42:127–52. doi: 10.1146/annurev-immunol-090222-102834 38060987

[6] Bai Z Lu Z Liu R Tang Y Ye X Jin M. Iguratimod restrains circulating follicular helper T cell function by inhibiting glucose metabolism via hif1alpha-HK2 axis in rheumatoid arthritis. Front Immunol. (2022) 13:757616. doi: 10.3389/fimmu.2022.757616 35720293 PMC9199372

[7] Liu R Wu Q Su D Che N Chen H Geng L. A regulatory effect of IL-21 on T follicular helper-like cell and B cell in rheumatoid arthritis. Arthritis Res Ther. (2012) 14:R255. doi: 10.1186/ar4100 23176102 PMC3674600

[8] Rao DA Gurish MF Marshall JL Slowikowski K Fonseka CY Liu Y. Pathologically expanded peripheral T helper cell subset drives B cells in rheumatoid arthritis. Nature. (2017) 542:110–4. doi: 10.1038/nature20810 PMC5349321 28150777

[9] Murray-Brown W Guo Y Small A Lowe K Weedon H Smith MD. Differential expansion of T peripheral helper cells in early rheumatoid arthritis and osteoarthritis synovium. RMD Open. (2022) 8:e002563. doi: 10.1136/rmdopen-2022-002563 36270740 PMC9594577

[10] Fortea-Gordo P Nuno L Villalba A Peiteado D Monjo I Sanchez-Mateos P. Two populations of circulating PD-1hiCD4 T cells with distinct B cell helping capacity are elevated in early rheumatoid arthritis. Rheumatol (Oxford). (2019) 58:1662–73. doi: 10.1093/rheumatology/kez169 31056653

[11] Zhang F Wei K Slowikowski K Fonseka CY Rao DA Kelly S. Defining inflammatory cell states in rheumatoid arthritis joint synovial tissues by integrating single-cell transcriptomics and mass cytometry. Nat Immunol. (2019) 20:928–42. doi: 10.1038/s41590-019-0378-1 PMC6602051 31061532

[12] Lucas C Perdriger A Ame P. Definition of B cell helper T cells in rheumatoid arthritis and their behavior during treatment. Semin Arthritis Rheumatol. (2020) 50:867–72. doi: 10.1016/j.semarthrit.2020.06.021 32896702

[13] Ding T Niu H Zhao X Gao C Li X Wang C. T-follicular regulatory cells: potential therapeutic targets in rheumatoid arthritis. Front Immunol. (2019) 10:2709. doi: 10.3389/fimmu.2019.02709 31849938 PMC6901970

[14] Aldridge J Andersson K Gjertsson I Hultgard Ekwall AK Hallstrom M van Vollenhoven R. Blood PD-1+TFh and CTLA-4+CD4+ T cells predict remission after CTLA-4Ig treatment in early rheumatoid arthritis. Rheumatol (Oxford). (2022) 61:1233–42. doi: 10.1093/rheumatology/keab454 PMC8889294 34009274

[15] Bocharnikov AV Keegan J Wacleche VS Cao Y Fonseka CY Wang G. PD-1hiCXCR5- T peripheral helper cells promote B cell responses in lupus via MAF and IL-21. JCI Insight. (2019) 4:e002563. doi: 10.1172/jci.insight.130062 PMC6824311 31536480

[16] Argyriou A Wadsworth MH 2nd Lendvai A Christensen SM Hensvold AH Gerstner C. Single cell sequencing identifies clonally expanded synovial CD4(+) T(PH) cells expressing GPR56 in rheumatoid arthritis. Nat Commun. (2022) 13:4046. doi: 10.1038/s41467-022-31519-6 35831277 PMC9279430

[17] Yabe H Kamekura R Yamamoto M Murayama K Kamiya S Ikegami I. Cytotoxic Tph-like cells are involved in persistent tissue damage in IgG4-related disease. Mod Rheumatol. (2021) 31:249–60. doi: 10.1080/14397595.2020.1719576 32023137

[18] Gong M Choi SC Park YP Zou X Elshikha AS Gerriets VA. Transcriptional and metabolic programs promote the expansion of follicular helper T cells in lupus-prone mice. iScience. (2023) 26:106774. doi: 10.1016/j.isci.2023.106774 37216123 PMC10197114

[19] Choi SC Titov AA Abboud G Seay HR Brusko TM Roopenian DC. Inhibition of glucose metabolism selectively targets autoreactive follicular helper T cells. Nat Commun. (2018) 9:4369. doi: 10.1038/s41467-018-06686-0 30348969 PMC6197193

[20] Kanno Y Vahedi G Hirahara K Singleton K O’Shea JJ. Transcriptional and epigenetic control of T helper cell specification: molecular mechanisms underlying commitment and plasticity. Annu Rev Immunol. (2012) 30:707–31. doi: 10.1146/annurev-immunol-020711-075058 PMC3314163 22224760

[21] Crotty S. Follicular helper CD4 T cells (TFH). Annu Rev Immunol. (2011) 29:621–63. doi: 10.1146/annurev-immunol-031210-101400 21314428

[22] Cannons JL Qi H Lu KT Dutta M Gomez-Rodriguez J Cheng J. Optimal germinal center responses require a multistage T cell:B cell adhesion process involving integrins, SLAM-associated protein, and CD84. Immunity. (2010) 32:253–65. doi: 10.1016/j.immuni.2010.01.010 PMC2830297 20153220

[23] Weinstein JS Herman EI Lainez B Licona-Limon P Esplugues E Flavell R. TFH cells progressively differentiate to regulate the germinal center response. Nat Immunol. (2016) 17:1197–205. doi: 10.1038/ni.3554 PMC5030190 27573866

[24] Yusuf I Kageyama R Monticelli L Johnston RJ Ditoro D Hansen K. Germinal center T follicular helper cell IL-4 production is dependent on signaling lymphocytic activation molecule receptor (CD150). J Immunol. (2010) 185:190–202. doi: 10.4049/jimmunol.0903505 20525889 PMC2913439

[25] Xin A Masson F Liao Y Preston S Guan T Gloury R. A molecular threshold for effector CD8(+) T cell differentiation controlled by transcription factors Blimp-1 and T-bet. Nat Immunol. (2016) 17:422–32. doi: 10.1038/ni.3410 PMC5779087 26950239

[26] Lu Y Ruan Y Hong P Rui K Liu Q Wang S. T-cell senescence: A crucial player in autoimmune diseases. Clin Immunol. (2023) 248:109202. doi: 10.1016/j.clim.2022.109202 36470338

[27] Lioulios G Mitsoglou Z Fylaktou A Xochelli A Christodoulou M Stai S. Exhausted but not senescent T lymphocytes predominate in lupus nephritis patients. Int J Mol Sci. (2022) 23:13928. doi: 10.3390/ijms232213928 36430418 PMC9694088

[28] Xiao W Wang RS Handy DE Loscalzo J. NAD(H) and NADP(H) redox couples and cellular energy metabolism. Antioxid Redox Signal. (2018) 28:251–72. doi: 10.1089/ars.2017.7216 PMC5737637 28648096

[29] Yi HS Kim SY Kim JT Lee YS Moon JS Kim M. T-cell senescence contributes to abnormal glucose homeostasis in humans and mice. Cell Death Dis. (2019) 10:249. doi: 10.1038/s41419-019-1494-4 30867412 PMC6416326

[30] Jiang Q Wang J Jiang H Li W Sun Y Shan Y. Competitive binding of transcription factors underlies flexibility of T peripheral helper cells and T follicular helper cells in SLE. Rheumatol (Oxford). (2022) 61:4547–57. doi: 10.1093/rheumatology/keac112 35191465

[31] Yoshitomi H Ueno H. Shared and distinct roles of T peripheral helper and T follicular helper cells in human diseases. Cell Mol Immunol. (2021) 18:523–7. doi: 10.1038/s41423-020-00529-z PMC8027819 32868910

[32] Wacleche VS Wang R Rao DA. Identification of T peripheral helper (Tph) cells. Methods Mol Biol. (2022) 2380:59–76. doi: 10.1007/978-1-0716-1736-6_6 34802122

[33] Gu-Trantien C Migliori E Buisseret L de Wind A Brohee S Garaud S. CXCL13-producing TFH cells link immune suppression and adaptive memory in human breast cancer. JCI Insight. (2017) 2:e91487. doi: 10.1172/jci.insight.91487 28570278 PMC5453706

[34] Liarski VM Kaverina N Chang A Brandt D Yanez D Talasnik L. Cell distance mapping identifies functional T follicular helper cells in inflamed human renal tissue. Sci Transl Med. (2014) 6:230ra46. doi: 10.1126/scitranslmed.3008146 PMC4129446 24695686

[35] Du F Yang LH Liu J Wang J Fan L Duangmano S. The role of mitochondria in the resistance of melanoma to PD-1 inhibitors. J Transl Med. (2023) 21:345. doi: 10.1186/s12967-023-04200-9 37221594 PMC10207731

[36] Barber DL Wherry EJ Masopust D Zhu B Allison JP Sharpe AH. Restoring function in exhausted CD8 T cells during chronic viral infection. Nature. (2006) 439:682–7. doi: 10.1038/nature04444 16382236

[37] Bai Z Liu R Liu J Zhang C Wang Z Qi J. Pathologically expanded peripheral CD4+PD-1+Foxp3– T-cell subset promotes B-cell hyperactivity in patients with rheumatoid arthritis. Rheumatol Autoimmun. (2024) 4:27–36. doi: 10.1002/rai2.12114

[38] Wei X Niu X. T follicular helper cells in autoimmune diseases. J Autoimmun. (2023) 134:102976. doi: 10.1016/j.jaut.2022.102976 36525939

[39] Lin J Yu Y Ma J Ren C Chen W. PD-1+CXCR5-CD4+T cells are correlated with the severity of systemic lupus erythematosus. Rheumatol (Oxford). (2019) 58:2188–92. doi: 10.1093/rheumatology/kez228 31180450

[40] Yoshitomi H. Peripheral helper T cells, mavericks of peripheral immune responses. Int Immunol. (2023) 36:9–16. doi: 10.1093/intimm/dxad041 PMC10823579 37788648

[41] Elahee M Mueller AA Wang R Marks KE Sasaki T Cao Y. A PD-1(high)CD4(+) T cell population with a cytotoxic phenotype is associated with interstitial lung disease in systemic sclerosis. ACR Open Rheumatol. (2024) 6:429–39. doi: 10.1002/acr2.11671 PMC11246828 38698736

[42] Seki N Tsujimoto H Tanemura S Kojima S Miyoshi F Kikuchi J. Cytotoxic Tph subset with low B-cell helper functions and its involvement in systemic lupus erythematosus. Commun Biol. (2024) 7:277. doi: 10.1038/s42003-024-05989-x 38448723 PMC10918188

[43] Martins C Rasbach E Heppt MV Singh P Kulcsar Z Holzgruber J. Tumor cell-intrinsic PD-1 promotes Merkel cell carcinoma growth by activating downstream mTOR-mitochondrial ROS signaling. Sci Adv. (2024) 10:eadi2012. doi: 10.1126/sciadv.adi2012 38241371 PMC10798567

[44] Cheng SC Quintin J Cramer RA Shepardson KM Saeed S Kumar V. mTOR- and HIF-1alpha-mediated aerobic glycolysis as metabolic basis for trained immunity. Science. (2014) 345:1250684. doi: 10.1126/science.1250684 25258083 PMC4226238

[45] Semenza GL. Targeting HIF-1 for cancer therapy. Nat Rev Cancer. (2003) 3:721–32. doi: 10.1038/nrc1187 13130303

[46] Zheng Z Cai Y Chen H Chen Z Zhu D Zhong Q. CXCL13/CXCR5 axis predicts poor prognosis and promotes progression through PI3K/AKT/mTOR pathway in clear cell renal cell carcinoma. Front Oncol. (2018) 8:682. doi: 10.3389/fonc.2018.00682 30723697 PMC6349755

[47] Diaz-Basilio F Vergara-Mendoza M Romero-Rodriguez J Hernandez-Rizo S Escobedo-Calvario A Fuentes-Romero LL. The ecto-enzyme CD38 modulates CD4T cell immunometabolic responses and participates in HIV pathogenesis. J Leukoc Biol. (2024) 116:440–55. doi: 10.1093/jleuko/qiae060 38466822

[48] Jiang P Zhao S Li X Hu S Chen S Liang Y. Dedicator of cytokinesis 8 (DOCK8) mutation impairs the differentiation of helper T cells by regulating the glycolytic pathway of CD4(+) T cells. MedComm. (2024) 5:e747. doi: 10.1002/mco2.747 39329018 PMC11424684

[49] Zhao M Wang Y Li L Liu S Wang C Yuan Y. Mitochondrial ROS promote mitochondrial dysfunction and inflammation in ischemic acute kidney injury by disrupting TFAM-mediated mtDNA maintenance. Theranostics. (2021) 11:1845–63. doi: 10.7150/thno.50905 PMC7778599 33408785

[50] Zhu M Min S Mao X Zhou Y Zhang Y Li W. Interleukin-13 promotes cellular senescence through inducing mitochondrial dysfunction in IgG4-related sialadenitis. Int J Oral Sci. (2022) 14:29. doi: 10.1038/s41368-022-00180-6 35718799 PMC9207030

[51] Dasoveanu DC Park HJ Ly CL Shipman WD Chyou S Kumar V. Lymph node stromal CCL2 limits antibody responses. Sci Immunol. (2020) 5:eaaw0693. doi: 10.1126/sciimmunol.aaw0693 32198221 PMC7490901

